# Fast and Enhanced MMW Imaging System Using a Simple Row Detector Circuit with GDDs as Sensor Elements and an FFT-Based Signal Acquisition System

**DOI:** 10.3390/s23031578

**Published:** 2023-02-01

**Authors:** Arun Ramachandra Kurup, Daniel Rozban, Amir Abramovich, Yitzhak Yitzhaky, Natan Kopeika

**Affiliations:** 1 Department of Electrical and Electronic Engineering, Ariel University, Ariel 40700, Israel; 2 Department of Electro-Optics and Photonics Engineering, School of Electrical and Computer Engineering, Ben-Gurion University of the Negev, Beer Sheva 84105, Israel

**Keywords:** glow discharge detectors, MMW imaging, THz sensors, quasi-optical design, focal plane array

## Abstract

The relatively high atmospheric propagation of millimeter-waves (MMW) was found to be one of the most critical reasons for the development of reliable sensors for MMW detection. According to previous research works, it has been already shown that incident MMW radiation on a glow discharge detector (GDD) can increase the discharge current. Hence, the electrical mode of detection can be employed to detect the presence of MMW radiation. In this article, a new design of a row detector using GDDs as pixel elements, and the influence of MMW incidence on GDD’s discharge current, were acquired using an elementary data acquisition (DAQ) platform. The DAQ system computes the averaged Fast Fourier Transform (FFT) spectrum of the time signal and returns the FFT results as magnitude based on the level of detection. An FFT-based signal acquisition proved to be a better alternative to the lock-in detection that was commonly used in MMW detection systems. This improved detection circuit provides enhanced noise filtering, thereby resulting in better MMW images within a short time. The overhead expense of the entire system is very low, as it can avoid lock-in amplifier stages that were previously used for signal enhancement. A scanning mechanism using a motorized translation stage (step motor) is involved to place and align the row detector in the image plane. The scanning can be carried out vertically to perform the imaging, by configuring the step motor after selecting the desired step size and position. A simplified version of the MMW detection circuit with a dedicated over-voltage protection facility is presented here. This made the detection system more stable and reliable during its operation. The MMW detection circuit demonstrated in this work was found to be a milestone to develop larger focal plane arrays (FPA) with very inexpensive sensor elements.

## 1. Introduction

Considering the fact that the atmospheric propagation of MMW/Terahertz (THz) waves is generally rather good, it is a desirable band for applications in various domains such as medicine, communication, and security [1,2] and there is a huge need for developing reliable and inexpensive MMW detectors [3]. The commercially available MMW detectors such as Schottky diodes, semiconductor MMW detectors, pyroelectric detectors, Golay cells, and microbolometers are expensive [4]. Despite the fact that the semiconductor MMW detectors can operate at room temperature with a higher sensitivity [1], their use is limited because they are highly sensitive to electrostatic discharge (ESD). In addition, they are very costly and their performance will be adversely affected and impaired during instances of incidence by high MMW radiation power. Even though Schottky diodes have a quick response time and similar noise-equivalent power (NEP), they are not employed for intense MMW radiation power as they are not too responsive at higher frequencies of the MMW spectrum [5].

It is known that the weakly ionized plasma (WIP) inside a glow discharge detector such as a neon lamp is highly sensitive to MMW/THz radiations. Additionally, when compared to the Schottky diode, it has a shorter response time [6]. In addition, GDDs can be used in the detection of high-power MMW radiation with both higher and faster responses [7]. Therefore, we use glow discharge detectors (GDD) as MMW/THz sensors in the detection circuit demonstrated here. This will also aid in simplifying the detection system, and obviously reduce the development cost of the focal plane array. The cost of MMW sensor elements can be a key factor in larger MMW focal plane arrays. Fortunately, each GDD sensor element costs only a few cents. The GDD is orders of magnitude less expensive compared to the price of other commercially MMW detectors [1].

Therefore, GDDs became attractive low-cost MMW/THz radiation detectors [3,4,5,6,7,8,9,10,11] and pixels in FPAs [12,13,14,15,16]. Here, we demonstrate a new implementation of a single row detector that can be expanded to any array size to form a dedicated FPA for MMW imaging. The working principle of the detection circuit here is based on the electrical mode of detection. In this mode of operation, due to the incidence of MMW radiation and the resultant enhanced cascade ionization process within the GDD [17], a change in the bias current [18] will occur and those variations can be detected using a suitable data acquisition platform. One of the major reasons to use the electrical detection method is its better responsiveness while using GDDs as detector pixels.

Several shortcomings exist in the previously developed FPAs that employ an electrical mode of detection [13,15], such as circuit complexity, damage to amplifiers due to voltage spikes during GDD switching, a need for external lock-in amplifiers, the long duration of image acquisition, and the requirement of additional signal conditioning elements. These motivated us to develop a much more reliable and simple detection circuit for MMW imaging. The digital algorithm used in this system enabled us to avoid an external lock-in amplifier stage, which was the most expensive part of our previous works. An FFT algorithm employed here was capable of extracting the magnitude of the detected signal particularly concentrated at 10 KHz modulation frequency. The dedicated amplifier IC, ADA4177-1 used here has a built-in capability to withstand sudden voltage spikes. This in turn improved the overall reliability of the electrical circuit. The step motor used for the translational movement was found to be more flexible for performing the over-sampling imaging process, and helped to increase the speed of the scanning process, thereby reducing the overall time to complete the imaging process. A fast image acquisition method using a simplified detection circuit model is the highlight of this work. In this work, we were able to reduce the cost of implementing the MMW detection and imaging process by utilizing the detection circuit used in the row detector. A small 8 × 8 GDD array is used to show obtained 100 GHz images.

The advantages of the proposed MMW detection over the previous low-cost GDD FPAs can be summarized as follows:

- The detection circuit provides better responsivity despite the high noise levels during GDD operation;
- It provides active overvoltage protection for the sensor circuit from the voltage spikes, which were common in previously developed detection circuits used in GDD-based FPAs;
- It makes an external lock-in amplifier unnecessary and, hence, reduces the complexity and the cost of the detection circuit;
- It facilitates the use of FFT-based filtering to perform the data acquisition in a more effective manner.

Such a simpler, more efficient, and inexpensive MMW detection circuit permits an extension to develop larger focal plane arrays that may be employed for real-time MMW imaging.

## 2. Method

The influence of MMW radiation incidence on GDD discharge current was measured using an elementary DAQ platform using NI USB-6341. The DAQ system computes the averaged FFT spectrum of the time signal and returns the FFT results as magnitude based on the level of detection. A mechanical system that has a motorized translation stage is used to move the row detector in the image plane. The scanning can be performed vertically to obtain the 8 × 8 pixel size image, by configuring the step motor after selecting the desired step size and position. A dedicated LabVIEW code was designed for the overall control and monitoring of the imaging system.

The electrical detection method measures the change in current through the GDD as a result of modulated radiation impact in on–off keying (OOK) modulation. A square wave signal of 0–5 V with 10 KHz frequency is the carrier signal to the MMW radiation source which enables the on–off keying, thereby modulating the radiation from the source. In this work, we demonstrated that a card with 8 channels can be used to convert an analog signal to a digital one while using the FFT algorithm. It permits good filtering of the noise and, hence, produces a better detection signal level even for extremely low radiation intensities.

### 2.1. Experimental Setup

**Figure 1:**
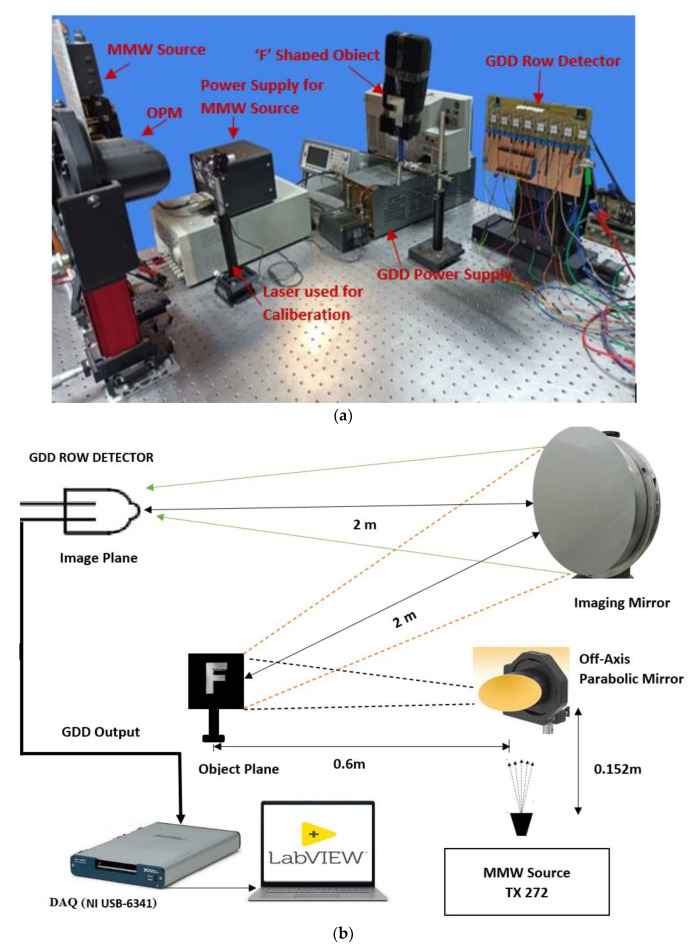
(**a**) Experimental setup of the MMW imaging system and (**b**) a schematic diagram of the system.

The experimental setup shown in Figure 1 is composed of an MMW source manufactured by Virginia Diodes Inc., Charlottesville, VA, USA (VDI TX272), a quasi-optic design based on one off-axis parabolic mirror (OPM), a reflecting/imaging mirror, a timer/counter type NI USB-6341 manufactured by National Instruments (NI), and row detector circuit composed of 8 GDDs of type N523 (International Light Inc., New York, NY, USA) as pixel/detector elements. The GDDs in the detection circuit were placed in the head-on configuration. Therefore, its total detection cross-section can be considered as a circle with a diameter of about 6 mm. Since sensitivity is strongest between the electrodes, the effective cross-section is at most about 3 mm in diameter, which is at least twice the electrode separation.

The MMW source is capable of radiating signals at a frequency range of 100 GHz, which was modulated using a 10 KHz square wave with an amplitude of 5 V peak to peak. The off-axis parabolic mirror placed in line to the horn antenna of the radiation source collects the MMW radiation and collimates the Gaussian beam to the metallic object placed in the object plane. The reflected radiation from the object was focused to a spherical mirror, which is capable of projecting the radiation to the image plane where the detection circuit was placed. Calibration and alignment were carried out using a laser in order to locate the GDD row detector at the reflective focal length of the spherical mirror. The entire imaging system design was based on a dedicated quasi-optical design which is detailed in Section 2.5. The detection circuit consists of GDDs which can serve as the pixel element of the focal plane array. The detection circuit is capable of recognizing the changes in the bias current of the GDD due to the incidence of MMW radiation. The detected analog signal is, in turn, connected to the data acquisition platform for further signal processing and generation of the grayscale image. The signal acquisition was implemented using an FFT-based algorithm that was incorporated in the LabVIEW code that was found to be more compatible with our detection system.

### 2.2. Design of the Row Detector

A row detector using 8 GDDs is employed here to detect the MMW radiation using the electrical detection method. A dedicated pre-amplifier, ADA4177-1, is attached to each GDD unit. The amplifier unit features outstanding precision amplifier robustness, providing input protection against signal fluctuations, as well as 70 dB of rejection for electromagnetic interference (EMI) at 1000 MHz. Thus, it can provide the necessary signal conditioning for later stages of the circuit. The ADA4177-1 uses active overvoltage protection to protect the devices from damage when the inputs are driven to a higher voltage. The ADA4177-1 not only protects the input from damage, but also reduces the input noise.

**Figure 2:**
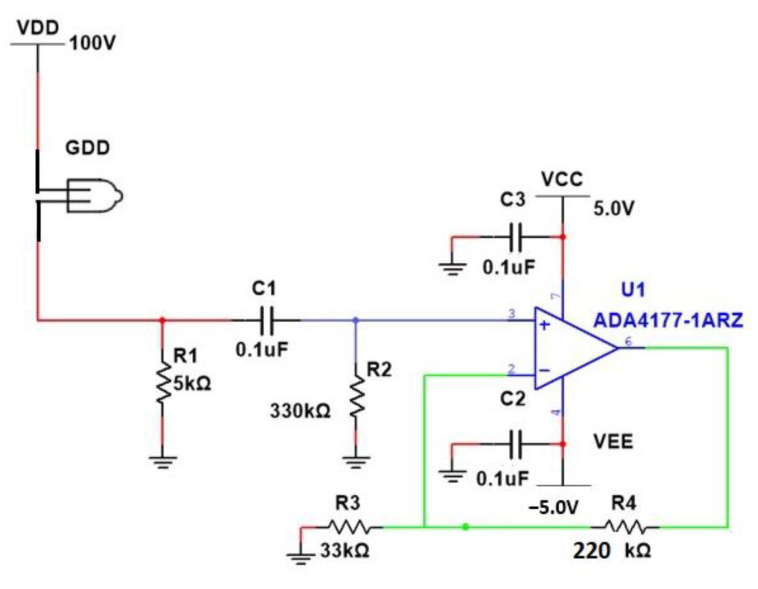
Detection circuit for a single GDD detector.

The electrical circuit used for one of the eight detectors is shown in Figure 2. Eight such circuits were assembled, and the outputs from all amplifiers were sampled simultaneously. The selected amplifier includes protection against voltage surges of 32 volts above the supply voltage of the amplifier. This dedicated row detector circuit is used to recognize the variation in the bias current of the GDDs due to the incidence of the reflected radiation from the metallic object placed in the object plane of the imaging system.

According to the datasheet of the GDD (N523), it requires an initial breakdown voltage of 100 V. In addition, the DC bias current in the non-radiation state is I_DC_ = 6 mA. When radiation is received by the detector, the MMW modulation generates an AC signal current through the detector, which is added to the DC bias current. The amplitude of the AC current is I_AC_ = 2 μA with an offset of 2 μA, so that an AC detection current varying between 4 μA and 0 A is obtained. The form of the AC current obtained is a square wave signal with a DC signal, as a result of the modulation signal given of the MMW radiation source. There is a filter stage in the row detector circuit so as to filter the DC signal and, therefore, the AC current is divided according to a current divider between resistor R1 and resistor R2.

After the filter stage, an amplifier section using an ADA4177 amplifier is employed, which can handle the fast and high voltage changes that are received at the input of the amplifier following the MMW radiation absorbed by the GDD detector. This amplifier is capable of dealing with rapid voltage fluctuations that are common in the operation of GDDs. The input voltage to the amplifier stage is very low and noisy, and this will be conditioned to a sufficient signal level by utilizing the gain of the amplifier. The amplified output signal is then fed to the analog to digital converter stage. The signal processing stage employs a digital algorithm based on FFT, which is explained in the succeeding section.

### 2.3. Measurement of the Minimum Detectable Signal

**Figure 3:**
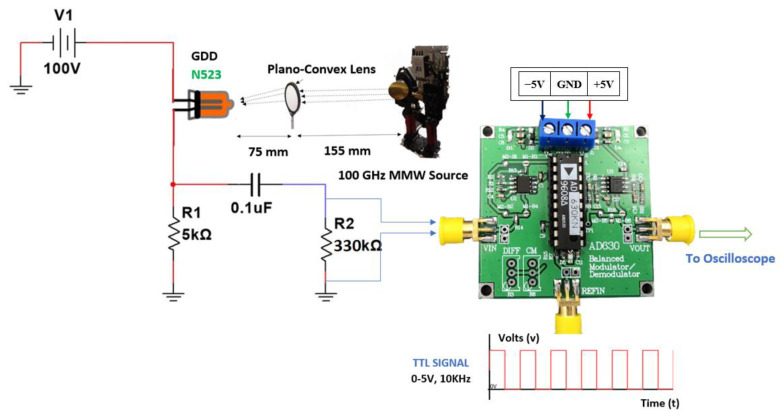
Experimental setup for measuring the minimum detectable signal using a lock-in amplifier integrated to a GDD detection circuit.

Initially, we examined the use of a lock-in amplifier module (AD630) in the output stage. Apart from the amplification, this stage can recover small signals even from the l00 dB interference noise. Measurements were carried out using the experimental setup shown in Figure 3 in order to find the minimum detectable signal from the GDD by integrating a lock-in amplifier into the circuit. This measurement was performed to make a comparison between the signal acquisition capability of the detection circuit using a GDD with a lock-in amplifier at the output stage, and one with our new proposed detection circuit used in building the row detector. A single GDD is employed here for measuring the minimum detectable signal. The detector was placed in the head-on configuration, which is similar to that in the row detector circuit. A plano-convex lens was used to focus most of the radiation properly on the GDD, so as to ensure maximum absorption of the MMW radiation. A User-Controlled Attenuator (UCA) supply voltage was varied from 0 to 5 V. It was found that the minimum detectable signal occurs when the UCA voltage level is at 4.6 V. At this UCA voltage level, the emitted power from the MMW radiation source is found to be 6 mW.

By configuring the DAQ at a sampling rate of 100 kHz, the FFT output is capable of acquiring the minimum detectable signal as accomplished with the lock-in amplifier. Finally, we have omitted the lock-in amplifier module as it can lead to an increase in the complexity of the circuit. It can also increase the time to acquire the final image as needed to pass the output signal of each GDD section to the lock-in amplifier. Otherwise, it is required to incorporate a lock-in amplifier module into each GDD section, which will increase the cost of the circuit. Thus, by using the same optical configuration, the proposed detection circuit without a lock-in amplifier module was placed under the exposure of MMW radiation and the maximum value of signal from an individual GDD in the row detector was measured with a peak-to-peak voltage level of 740 mV.

### 2.4. Scanning Mechanism Using a Step Motor

In the detection system, a scanning mechanism using a step motor on which the row detector was mounted, as shown in Figure 1a, was also incorporated for performing the movements of the row detector in horizontal and vertical directions to collect the MMW radiation reflected from the spherical mirror to the GDDs in the row detector. The row detector will be fixed on the mount of the step motor setup. Drivers of the step motor system are suitable for interfacing with LabVIEW software using a motor controller. The operation of the step motor is controlled by the LabVIEW code, through which the step sizes can be precisely provided. Thus, we were able to move the row detector in both horizontal and vertical directions in the image plane.

### 2.5. Quasi Optical Design

A dedicated quasi-optical design, as indicated in Figure 1b, is used in these experiments [19]. The imaging system includes a 100 GHz source, a collimating off-axis parabolic mirror (OPM), an imaging mirror, and a focal plane array (FPA). In this setup, we used an OPM of 152 mm focal length and aperture of 100 mm, and a spherical imaging mirror of 1000 mm focal length and aperture of 500 mm. An initial alignment of the quasi-optical setup was performed with the help of a laser source instead of the MMW radiation transmitter. The positions of quasi-optical dishes were adjusted so as to assure maximum focus of MMW signal on the GDD.

The Gaussian beam approximation was used to calculate the beam waist using Equation (1), and the total power collected by the GDD cross-section is calculated using Equation (2).

(1) $$\omega \left(z\right)=\omega_{0}\sqrt{1+\left(\frac{z}{z_{R}}\right)},{\;z}_{R}=\frac{{\pi \omega }_{0}}{\lambda }\;$$

(2) $${P=P}_{0}\left({1\;-\;e}^{-\;\frac{D^{2}}{2\omega {\left(z\right)}^{2}}}\right),\;$$

where ω(z) is the beam waist, z is the distance involved in propagation, λ is the MMW/THz wavelength, Z_R_ is the Rayleigh range of a Gaussian beam, P_0_ is the total beam power emitted from the MMW radiation source, and D is the effective diameter of the GDD sensor element.

According to the nominal horn specifications from the manufacturer of the radiation transmitter, the MMW beam waist radius is the input of the conical horn antenna and is fixed to the value ω_0_ = 6.2 mm. The MMW/THz radiation source was placed at the focal point of the OPM, and the detection circuit was placed at the focal point of the spherical imaging mirror. The approximate beam waist at a given position in the quasi-optical setup can be estimated using the transformation of the complex beam parameter q_1_ at the output of the MMW source (W = 6.2 mm and R = ∞) by the ABCD transfer matrix for paraxial rays. Using Equation (3), the complex beam parameter q_2_ at that given position can be calculated. From the value of q_2_, the beam waist (W) and the beam radius of curvature (R) at that given position is then calculated using Equation (1).

(3) $$\left[\begin{array}{cc} A & B\\ C & D \end{array}\right]=\left[\begin{array}{cc} 1 & d_{3}\\ 0 & 1 \end{array}\right]\left[\begin{array}{cc} 1 & 0\\ -1/F_{M} & 1 \end{array}\right]\left[\begin{array}{cc} 1 & d_{2}\\ 0 & 1 \end{array}\right]\left[\begin{array}{cc} 1 & 0\\ -1/F_{\mathrm{OPM}} & 1 \end{array}\right]\left[\begin{array}{cc} 1 & d_{1}\\ 0 & 1 \end{array}\right]$$

(4) $$q_{2}=\frac{{\mathrm{Aq}}_{1}+B}{{\mathrm{Cq}}_{1}+D}$$

(5) $$\frac{1}{q_{2}}=\frac{1}{R}-\frac{i\lambda }{{\pi W}^{2}}$$

The parameters in Equation (3) are as follows: d_1_ is the distance between the transmitter and the OPM, F_OPM_ is the focal length of the OPM, F_M_ is the focal length of the imaging mirror, d_2_ is the distance between the OPM and the object, and d_3_ is the distance between the imaging mirror and the FPA.

### 2.6. Signal Acquisition Method Using FFT

Fast Fourier Transform is employed here to acquire the data from the DAQ. The FFT Spectrum (Mag-Phase) Virtual Instrument (VI) is particularly employed because of its capability to efficiently calculate the magnitude and phase of different frequency components in time-varying signals. The relevant module computes the averaged FFT spectrum of the time signal. The VI returns the acquired results as an array of magnitudes of the averaged FFT spectra, one per input waveform. The computation of the magnitude and phase is performed in three stages; computing the FFT of the time signal, averaging the current spectrum of the time signal with the FFT spectra computed by the VI since the last time the averaging process was reset, and then yielding the magnitude and phase of the averaged spectrum. The FFT-based algorithm employed here could extract the magnitude of the detected signal, which is concentrated particularly in the 10 KHz frequency component at which the MMW radiation is modulated in the detection system. This enhances the performance of the detection system by filtering the noise components accompanied by other frequency components.

### 2.7. Simulation Data vs. Experimental Data

**Figure 4:**
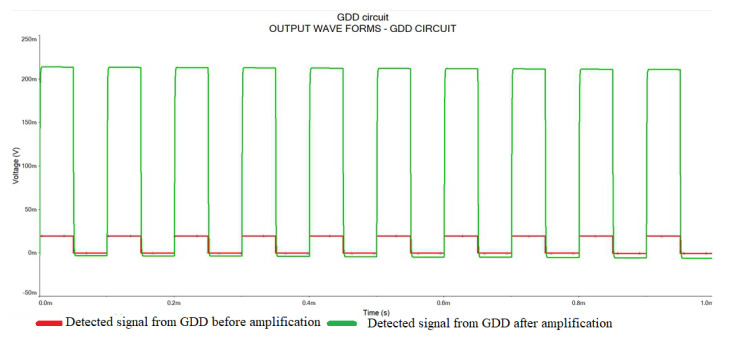
Output waveforms from the simulation of the GDD circuit.

The ADA4177-1 was used in the electrical detection module to amplify the input electrical signal that has been generated from the electrode of the GDD due to the incidence of MMW radiation on the FPA. As shown in Figure 4, the simulation results from the SPICE simulator of the said electrical circuit show an excellent voltage gain in the range of 10. However, the actual experimental results showed some decrease in the gain level mentioned in this section.

**Figure 5:**
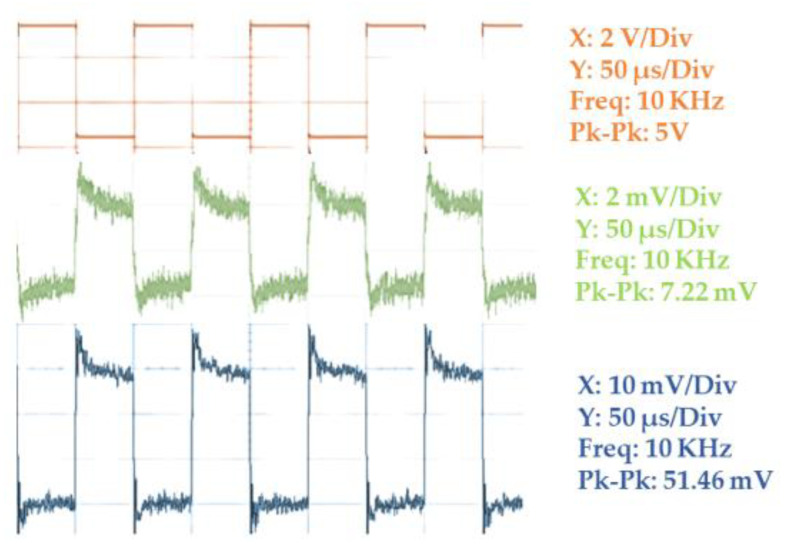
Output waveform from an individual GDD in the row detector circuit before amplification, and after amplification.

The simulation results indicated a voltage gain of 10 by the amplifier stage built by ADA4177-1. This result is good for better MMW detection by the electrical detection method by providing stronger signals to the data acquisition module. It was noticed that a voltage gain of 7.14 is achieved by this circuit. The output waveforms received from the detection circuit before and after the amplifier stage are indicated in Figure 5. In the experimental system, a modulating signal of 10 KHz frequency which has an amplitude of 5 V is used. This square wave will provide an on–off keying which will enable us to observe the detection of MMW/THz radiation by the detector circuit.

In Figure 5, the modulating signal is the reference signal that creates two instances for MMW radiation ON and MMW radiation OFF. Accordingly, the analog signal voltage output from the detection circuit can be observed on the oscilloscope. For comparison purposes, we have taken outputs from two points from one of the GDDs in the row detector; one before the amplification stage and the other from the amplified end. Envelope detection was observed when the modulating voltage was taken as the carrier signal of MMW radiation. The decrease in the voltage gain is mainly due to the losses in the circuit, and the propagation loss associated with the MMW transmission.

### 2.8. Imaging by Oversampling

An enhanced imaging result is obtained by performing the oversampling. Previous work already proved that image-based shifting-oversampling can improve the MMW images [20]. This is achieved by acquiring sub-pixel values by moving the row detector with 8 GDDs horizontally (step size = 3 mm) and vertically (step size = 4 mm), so that we obtain 16 rows and 16 columns instead of 8 each. The step movement was executed by using the Linear Stage Motor Control (step motor). The holding/waiting time given for both horizontal and vertical step movement was 1000 ms. Therefore, in 32 s we effectively obtain a 16 × 16 pixel image. This is an improvement, while considering the scanning time employed in the previous works [14,20], where the movements of the FPA were performed manually rather than via the step motor employed in this work. The GUI provided by the manufacturer of the step motor was used for controlling the movement of the step motor.

## 3. Results and Analysis

### 3.1. Using the GDD Row Detector

The row detector circuit built on a PCB was mounted on the step motor using a holder that helps to align and hold the board properly for the imaging experiment. A metallic ‘F’ shaped object was used for the imaging experiment. The outputs from each amplifier of the eight GDD circuit sections were connected to the eight analog input channels of the DAQ in order to acquire the signals due to MMW detection by the GDDs.

**Figure 6:**
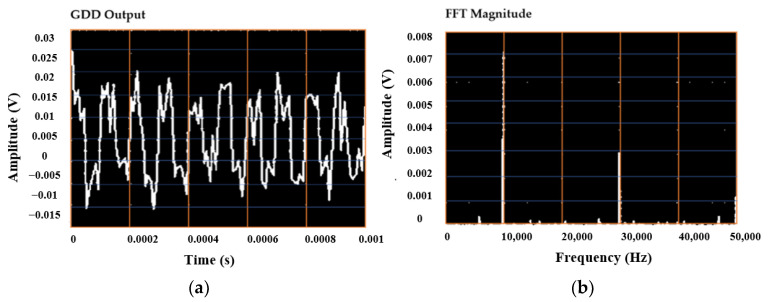
(**a**) Output waveform from an individual GDD in the row detector and (**b**) magnitude of the FFT of that GDD output signal.

The data acquisition module used here to acquire the signals from the GDD detection circuit is NI-USB 6341, which was controlled by a LabVIEW code. A user-friendly front panel view was also developed for the overall control and monitoring of the imaging system. The detected signal and the corresponding FFT magnitude, as shown in Figure 6a,b, demonstrate the output of an individual GDD in the row detector due to the incidence of MMW/THz radiation during the imaging. The NI-USB 6341 DAQ has sixteen single-ended or eight differential analog input channels with a maximum single-channel sampling rate of 500 k samples/s, and has an ADC resolution of 16 bits. The DAQ was configured at a sampling rate of 100 kHz and, because of the bottleneck of sampling rate by the DAQ, the DAQ was configured into two sequences so as to acquire signals from the eight GDDs of the row detector.

**Figure 7:**
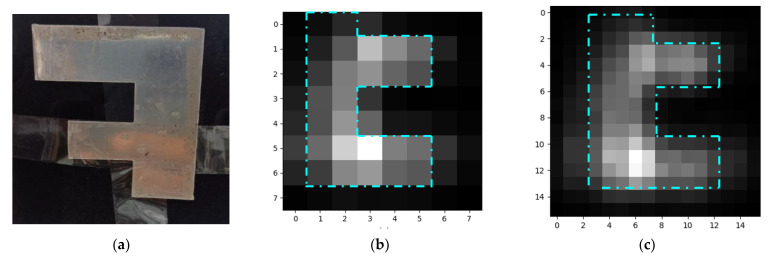
(**a**) ‘F’-shaped metallic object, (**b**) 8 × 8 Grayscale image, and (**c**) the oversampled 16 × 16 Grayscale image.

The reflected MMW radiation from the metallic ‘F’-shaped object shown in Figure 7a was detected by the GDDs in the row detector, and the corresponding pixel values are analyzed to create a grayscale image as indicated in Figure 7b. By performing oversampling, a 16 × 16 grayscale image was generated, as shown in Figure 7c. The image is inverted, as expected from geometrical optics.

The quality of the imaging results is mainly limited by the pixel resolution of the focal plane array and by the inherent diffraction limit of the GDD-based MMW imaging systems [14].

### 3.2. Calculation of NEP and Responsivity of the System

The noise levels of the detection circuit were measured using a spectrum analyzer and found to be 2.1 *×* 10^−6^ V/√Hz. The bandwidth of the amplifier used in the detection circuit is calculated from the frequency response and found to be 60 kHz.

A calculation of the system noise-equivalent power (NEP) was carried out, using

(6) $$NEP=\frac{V_{n}}{R_{e}\;\;\sqrt{B}}$$

where *V_n_* is the noise voltage, *B* is the bandwidth, and *R_e_* represents the responsivity of the detection system.

The GDD region most responsive to the MMW radiation is that between the electrodes. Since electrode separation is about 1 mm, we take the effective detection region to be a cylinder of about 3 mm diameter, which is half of the diameter of the GDD in the face-on configuration. Since only one quarter of the MMW power incident on the GDD reaches this GDD detection cross-section, the total MMW radiation power incident on this cross-section was calculated to be 34 mW. The detected signal from the amplifier output was measured to be 740 mV. Hence, the responsivity for the detection system, *R_e_*, can be taken as 21.8 V/W. By substituting these values into Equation (6), the NEP can be calculated as

$$\mathrm{NEP}=\frac{2.1\times 10^{-6}}{21.8\times \sqrt{60\;\times \;10^{3}}}=3.9\times 10^{-10}\;W/\surd \mathrm{Hz}$$

The measured value of NEP is about an order of magnitude better than the previous experimental result [3]. Additionally, the value of minimum detectable signal was calculated using the equation

(7) $$NEP=\frac{P_{s-min}}{\sqrt{B}}$$

where $P_{s-min}$ is the minimum detected signal power.

According to Equation (7), the minimum detected signal power of the system was found to be about 0.95 nW (when multiplying the NEP with the square root of the bandwidth of the amplifier used in the detection circuit). By positioning parabolic reflectors behind each GDD to focus all the incident radiation onto the region between the electrodes, the detection efficiency can be improved according to the diameter of such parabolic reflectors.

### 3.3. Comparison of Imaging Results Obtained Using GDD and Schottky Diode

It was explicitly proved in [21] that the commercial neon lamps or GDDs can be used as an effective MMW detector after comparing the imaging results obtained from the Schottky diode and GDD using the same MMW imaging system configuration. Even within the limits of aperture for the receiving, the proposed FPA using GDDs can produce images with about the same quality as obtained with the Schottky diode detector assembly. One of the major challenges in signal acquisition using GDD based FPA is that the detection characteristics may vary between individual GDDs, even though they are from the same manufacturer. However, considering the cost of components, the MMW detection system using the Schottky diode-based detector is far more expensive than the FPAs constructed using the GDDs. The quality of the imaging results, in both cases, is mainly limited by the low 8 × 8 focal plane array pixel resolution, causing a severe pixelation effect and a reduction in contrast.

In [6], the output signals from a single WIP based detector, whose core element was a GDD, and that from a single Schottky diode detector were compared using mechanical scanning to generate high resolution images, as in airport scanners. There was no difference in image quality between the GDD and Schottky diode images. Additionally, the output from the Schottky diode detector will be distorted due to its non-linear responsivity at higher MIMW/THz radiation power [6]. Here, we are concerned with simplifying and facilitating the detection process for focal plane arrays in employing the inexpensive but more responsive GDDs as the detector elements.

## 4. Conclusions

The present work envisages a neoteric method for MMW detection and thereby enhances a simple and inexpensive imaging technique. The electrical detection method that uses a Fast Fourier Transform (FFT) for data acquisition gains a fast readout system and delivers better imaging results. This FFT based system was designed to extract only the 10 KHz frequency component, as the MMW radiation source was modulated using that frequency. The rest of the frequency components were neglected and, thus, it improved the performance of the imaging system. Additionally, the FFT algorithm can perform an effective noise-filtering process. The signal acquisition from the detection circuit was performed at a sampling rate of 100 KHz for 20 k samples. This configuration enabled the row detector circuit to achieve a better detection capability, even in the absence of a lock-in amplifier. The GDD as a sensor element is very sensitive to MMW radiation and can be used as a pixel in an FPA, from which the pixel readout can be performed using a data acquisition platform. A parallel signal readout that can simultaneously acquire the signal from the FPA can significantly reduce the time taken to record an MMW image using this row detector. However, a trade-off should be kept in this regard as the scanning mechanism employed here using the step motor has limited velocity in its movement.

During the experiments, it was possible to develop a 16 × 16 image with a frame acquisition time of 32 s. This frame acquisition time can be easily improved by incorporating larger focal plane arrays, thereby providing a parallel readout system. It is also possible to generate enhanced MMW images with the same experimental setup by providing more waiting time between two consecutive step movements. The image-capturing speed can be improved by increasing the sampling frequency and the modulation frequency of the MMW radiation source. By doing so, it is possible to generate a 16 × 16 image within a few seconds. The same circuit design can be expanded to form a higher-order FPA, to form a matrix. Through this, it is possible to avoid the translational stage so that the MMW image can be generated within a second by using the required data acquisition platform that can acquire the signals from all pixels simultaneously. An added benefit is a sensitivity improvement by about an order of magnitude higher compared to what was achieved through focal plane array imaging using GDD as sensor elements [12]. The resolution of the imaging system demonstrated here can be improved by incorporating more than 8 × 8 pixels. Here, we showed a proof of concept of a simple row detector which can be expanded to form larger focal plane arrays for attaining improved image quality.

## 5. Future Work

Future works involve the development of a compact 32 × 32 FPA with this signal readout technology. The construction and development of a 32 × 32 focal plane array without any moving parts can be used for a fast MMW imaging system that can generate a real-time image. The provision to add reflectors to individual GDDs in the FPA is also under consideration. The inclusion of reflectors will help to focus more MMW radiation that has been reflected from the imaging mirror, which will pave the way to acquire an enhanced imaging result. The proposed expansion of focal plane arrays can be used for security applications such as the detection of concealed objects, as well as any real-time MMW imaging application.

## Data Availability

Not applicable.
